# Heavy Metal Pollution and Risk Assessment of Surface Dust in the Arid NW China

**DOI:** 10.3390/ijerph192013296

**Published:** 2022-10-15

**Authors:** Xiuyun Yang, Mamattursun Eziz, Adila Hayrat, Xiaofei Ma, Wei Yan, Kaixuan Qian, Jiaxin Li, Yuan Liu, Yifan Wang

**Affiliations:** 1 College of Geographical Science and Tourism, Xinjiang Normal University, Urumqi 830054, China; 2 China State Key Laboratory of Desert and Oasis Ecology, Xinjiang Institute of Ecology and Geography, Chinese Academy of Sciences, Urumqi 830011, China; 3 Research Centre for Ecology and Environment of CA, Chinese Academy of Sciences, Urumqi 830011, China; 4 School of Geographic Sciences, Xinyang Normal University, Xinyang 464000, China; 5 Key Laboratory of Smart City and Environment Modelling of Higher Education Institute, College of Resources and Environment Science, Xinjiang University, Urumqi 830046, China

**Keywords:** urban surface dust, heavy metals, positive matrix factorization, health risks, oasis city

## Abstract

High concentrations of heavy metals (HMs) in urban surface dust (USD) can be extremely hazardous to urban ecology and human health. Oasis cities are located at the edge of deserts and are more exposed to salt/sandstorms, and they face a significantly higher accumulation of USD than wet or semi-humid areas. However, systematic studies on the pollution and risk assessment of HMs in USD in oasis cities have rarely been conducted. This study systematically analyzed the enrichment status, spatial distribution, pollution levels, health risks, and sources of HMs in USD in a typical oasis city (Changji city). The results showed that the average concentrations of Pb, Ni, As, Cd, Hg, and Cu in the USD of Changji city were 46.83, 26.35, 9.92, 0.21, 0.047, and 59.33 mg/kg, respectively, and the results of the pollution index evaluation showed moderate Pb, Hg, and Cu pollution, mild Cd pollution, and no Ni or As pollution. The spatial distribution of HM concentrations in the USD was substantially heterogeneous. High values of Pb, Hg, and Cu concentrations were mainly observed in areas with relatively intensive transportation and commercial activities, and high values of Cd and Ni were observed in industrial areas. The health risk assessment showed that HMs do not pose non-carcinogenic risks to humans at their current level, but they pose a carcinogenic risk to children, with As contributing the largest carcinogenic and non-carcinogenic risks. The source identification of HMs showed that the main pollution of HMs were traffic sources for Pb and Cu, industrial sources for Ni and Cd, natural sources for As, and coal-fired sources for Hg. According to the results of the quantitative analysis with the positive matrix factorization, the contribution of pollution sources followed this order: industrial sources (31.08%) > traffic sources (26.80%) > coal-fired sources (23.31%) > natural sources (18.81%).

## 1. Introduction

Urban surface dust (USD) is a pollutant consisting of one or more organic and inorganic particles with strong environmental indicator effects, and its sources are mainly influenced by a combination of natural and anthropogenic factors, such as industrial emissions, transportation, urban construction, municipal emissions, atmospheric deposition, and soil erosion [1,2,3,4,5,6]. USD with high concentrations of heavy metals (HMs) poses serious risks to urban ecology and human health [7,8]. In recent years, with rapid urbanization and industrialization, a large number of people have moved from rural areas to cities, leading to an explosive increase in the type or quantity of pollutants in the urban ecosystem. Consequently, the balance of the urban ecosystem has deteriorated, thus impairing the self-healing capacity of the ecosystem and eventually leading to a variety of pollution problems in the urban ecosystem [9,10]. USD generally has a loose structure, tiny particles, and a relatively smooth attachment surface, and it can enter the atmosphere through resuspension under external disturbances. In addition, some dust can be transported to areas far from the pollution source through atmospheric turbulence and the prevailing winds [11,12,13]. Meanwhile, some particles suspended in the atmosphere may irritate the nose, throat, and eyes, and dust particles less than 2.5 µm in diameter can enter the lungs directly, thus posing serious health risks [14,15]. In addition, dust suspended in the atmosphere continuously enters urban ecosystems under the influence of both gravity and precipitation [16,17,18]. Moreover, USD can enter urban water bodies through the surface runoff formed by precipitation and municipal road cleaning, thus leading to the continuous enrichment of many toxic pollutants in water bodies and eventually threatening the growth and reproduction of aquatic plants and animals [19,20,21]. In addition, many HMs accumulate in the human body over long periods of time through accidental ingestion, inhalation, and dermal contact, thus causing irreversible damage to human health [22,23,24]. Therefore, USD can serve as a comprehensive indicator of the quality of the urban ecological environment [25,26].

Many methods have been used to quantify the pollution levels, ecological risks, health risks, and sources of HMs in USD [27,28,29,30]. The pollution index method is often used to quantitatively analyze the pollution levels of HMs in USD because of its simple calculation and relatively accurate assessment results. For example, studies on the pollution levels of HMs in USD in Zhengzhou (China), Surgut (Russia), Bandar Abbas (Iran), and Sonbhadra (India) using the geoaccumulation index [31], enrichment factor [32], Nemerow pollution index [33], and Tomlinson pollution load index [34], respectively, have revealed the HM contamination of urban ecosystems. Various models have been developed for assessing the risks of HMs to humans, including BCA (risk-based corrective action) [35], the CLEA (Contaminated Land Exposure Assessment) [36], and the HHRE (Human Health Risk Evaluation) [8]. These models require the input of contaminant exposure parameters and environmental parameters, which improves the accuracy of the assessment results [37]. Therefore, the above models are often used to quantify the health risk of HMs in surface dust to humans, among which the BCA model is the most widely used. Zhang et al. [38], Chen et al. [39], and Xiao et al. [40] used the BCA model to evaluate the health risks of HMs in USD from different cities in China and showed that HMs pose some health hazards to humans. Roy et al. [15] quantitatively analyzed HMs in USD on a global scale and found that children in Asia and Australia are already at the edge of susceptibility to non-carcinogenic health risks from Pb in USD. Children are generally more exposed to HMs from USD than adults [41,42]. As different cities have large differences in many natural and socioeconomic aspects, such as topographical conditions, climatic characteristics, soil types, urban scale, and industry types, the main sources of HMs are variable [43,44,45,46]. Therefore, the accurate source identification of HMs in USD is the key to reducing the risk of HMs to the urban ecological environment and human health [47]. The diffusion model and the receptor model are the main methods used for the quantitative analysis of HMs pollution sources [48]. Among them, the receptor model has been widely used because it does not depend on the emission conditions, topography, and meteorological data of the pollution sources compared to the diffusion model and the diffusion process of pollutants need not be tracked, thus avoiding the uncertainty of the input data of the diffusion model [49,50,51,52].

Oasis cities are built on a large-scale desert background substrate, and their urban development is largely constrained by the distribution characteristics of the water system [53,54]. In addition, oasis cities have a dry climate, sparse vegetation, and exposed surfaces due to a low precipitation and high evaporation, and their urban ecological environment is vulnerable to dust storms under the effects of human activities and atmospheric disturbances [26,55]. Luo et al. [56] quantitatively analyzed the contamination and health risk of HMs in the dust of typical oasis cities from sandstorms and found HM contamination derived from anthropogenic activities; moreover, exposure through ingestion poses the greatest health risk to humans. Yu et al. [57] reported that the dust concentration in the atmosphere of the oasis cities (Kashgar and Hotan) on the edge of the Tarim Basin was higher than that of Urumqi and Lanzhou, but the concentration was similar to that of Jeddah and Mecca in Saudi Arabia. In addition, dust pollution in oasis cities is one of the major contributors to a reduced human life expectancy [58]. Overall, the long-term enrichment of HMs in the USD of oasis cities has had adverse effects on the urban ecological environment and human health.

An assessment report on global dust storms found that about 75% of the global dust originated from natural sources, with dry climates, sparse vegetation, and loose soil in arid areas becoming the largest production areas of sand sources [59,60,61]. Oasis cities are located at the edges of deserts, and the frequency and intensity of dust storms are usually higher than those in humid areas, which has a negative impact on urban ecology and residents’ health [62,63,64]. A study of HMs in air dust from a typical oasis city in Iran found that most of the HMs in Zabol air dust had a high bioaccessibility and the associated HMs produced carcinogenic and non-carcinogenic risks for both adults and children that were greater than the safety threshold [65]. This indicates that HM contamination in Zabol air dust has posed some risk to human health. Another large oasis city in Iran (Ahvaz) has a high contamination of HMs in surface dust, with Hg, Pb, Mn, V, and Cr exhibiting higher health risks to residents of both residential and industrial areas [66]. The concentration of particulate-bound Hg in the USD of different oasis cities (Ahvaz, Asaluyeh, and Zabol) is associated with industrial development and oil- and gas-related activities [67]. In addition, children pose a considerable non-carcinogenic health risk to their bodies through the inhalation of indoor dust in Ahvaz. Overall, HMs in the USD of oasis cities are significantly harmful to urban ecology and human health [68,69]. Therefore, in this study, a typical oasis city (Changji city) was selected as the study area, and the enrichment status, spatial distribution, pollution level, health risk, and sources of HMs in USD were quantitatively analyzed in order to provide data support for monitoring ecological safety and human health in oasis cities in arid regions. The primary objectives of the research are: (1) to analyze the concentration of HMs in USD and their pollution levels; (2) to draw a 3D map of HM concentrations and pollution levels; (3) to assess the carcinogenic and non-carcinogenic risks of HMs to humans; and (4) to quantitatively analyze the sources of HMs in USD.

## 2. Materials and Methods

### 2.1. Study Area

**Figure 1:**
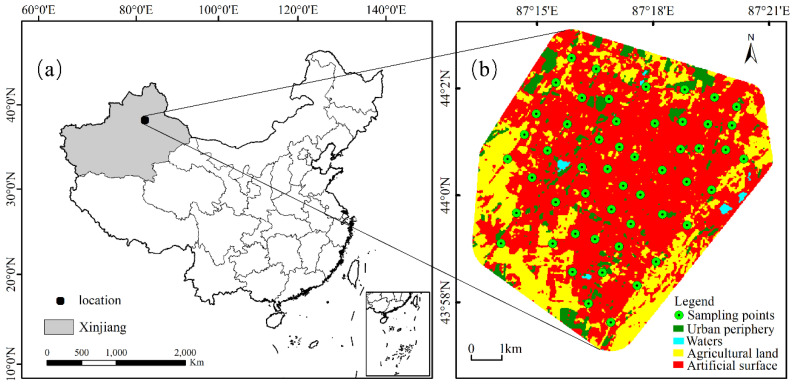
A map of the study area and sampling points. (**a**) Location of Xinjiang in China; (**b**) spatial distribution map of sampling sites in Changji City.

Changji city is a typical oasis city in the arid northwest of China [70]. It is one of the core cities of the northern slope economic belt of the Tianshan Mountains and the Silk Road Economic Belt in Xinjiang, NW China. The study area is located in the northern piedmont plains of the Tianshan Mountains, on the southern edge of the Junggar Basin. It covers 77.43 km^2^ and is located at 87°13′17″87°21′05″ E and 43°57′04″44°03′06″ N, with elevations ranging from 480 m to 593 m (Figure 1). The city has a continental desert climate with average annual air temperatures, precipitation, and evaporation of 6.8 °C, 190 mm, and 1780 mm, respectively, and prevailing northwest and southeast winds throughout the year [71]. In addition, Changji enjoys superior geographical conditions and is known as the “throat of the Western Regions”. It is the golden channel and a bridgehead linking China to Central Asia and Europe [72,73]. The area has variable but huge quantities of proven mineral resources. The land use type in the study area is dominated by construction land, agricultural land, and urban periphery. The resident population in the urban area of Changji was approximately 420,000 in 2019, and the regional economic growth is dominated by secondary and tertiary industries.

### 2.2. Sample Collection, Analysis, and Quality Control

In October 2019, 52 USD samples were collected from major urban areas in Changji which had been considerably affected by anthropogenic activities, using the methodology described in “NY/T 395-2012” [74] (Figure 1). In order to avoid human contamination, the dust samples were collected using non-metallic instruments such as brushes and dustpans. To ensure the representativeness of the samples, each sample was mixed with five subsamples within the preset sample point range of 50 m × 50 m. All samples were air-dried in the laboratory and weighed 0.500 g after passing through 0.15 mm of nylon mesh. Next, the samples were digested by an HCl-HNO_3_-HF-HClO_4_ solution [75]. The duplicate and blank samples were set according to the Chinese national standards (GSS-12) for quality control. Finally, the concentrations of Pb, Ni, and Cd were measured using an atomic absorption spectrometer (GFAAS; SOLAAR-M6, Thermo Fisher Scientific Inc., Waltham, MA, USA), the concentrations of As and Hg were measured using an atomic fluorescence photometer (BAF-2000, Baode Instrument Co., Beijing, China), and the concentration of Cu was measured using an inductively coupled plasma (ICP-5000, Focused Photonics Inc., Hangzhou, China) emission spectrometer [76]. The Ni, As, Hg, and Cu testing standards are “GB/T 17139-1997” [77], “GB/T 22105.2-2008” [78], “GB/T 22105.1-2008” [79], and “GB/T 17138-1997” [80], respectively. The determination of lead and cadmium was done according to the standard “GB/T 17140-1997” [81]. The detection limits of Pb, Ni, As, Cd, Hg, and Cu were 0.10, 0.4, 0.01, 0.06, 0.002, and 0.01 mg/kg, respectively, and their recoveries ranged from 95.16% to 106.55%.

### 2.3. Pollution Assessment of HMs

The Nemerow comprehensive pollution index (*NPI*), which is based on a single pollution index, was applied to quantify the pollution level of HMs. This method provides scientific and reasonable pollution evaluation results by taking into account the average pollution level of all participating elements as well as the main contaminants in the quantitative assessment of the pollution status of HMs [82]. It was calculated using the following Equation:

(1) $$P_{i}=C_{i}/S_{i}$$

(2) $$NPI=\sqrt{\left(P_{i\max}^{2}+P_{i\mathrm{ave}}^{2}\right)/2}$$

where *P_i_* and *C_i_* indicate the single contamination index and the concentration of element *i*, respectively, and *S_i_* indicates the background value of element *i* in the soils of Xinjiang [60]. *P_i_*_max_ and *P_i_*_ave_ indicate the maximum and average values of *P_i_*, respectively. *P_i_* and *NPI* classification standards refer to [83].

### 2.4. Analysis of the Sources of HMs

The positive matrix factorization (PMF) receptor model has been widely used to quantify the contribution of pollution sources, which decomposes the concentration data matrix *X* (*m* × *n*) consisting of m samples with n elements into a factor score matrix *g* (*m* × *p*), a factor loading matrix *f* (*p* × *n*), and a residual matrix *e* (*m* × *n*), and the main computational Equations are as follows [84]:

(3) $$X_{i}=\overset{p}{\underset{k=1}{\sum }}g_{ij}f_{ij}+e_{ij}$$

(4) $$Q=\overset{n}{\underset{i=1}{\sum }}\overset{m}{\underset{j=1}{\sum }}\left(\frac{\delta_{ij}}{u_{ij}}\right)$$

(5) $$U_{nc}=\{\begin{array}{c} 5/6\times MDL\\ \sqrt{{\left(\delta \times c\right)}^{2}+{\left(MDL\right)}^{2}} \end{array}\begin{array}{c} C\leq MDL\\ C>MDL \end{array}$$

where *X_ij_* represents the measured concentration matrix of element *i* in sample *j*, *g_jk_* represents the contribution matrix of source k to sample *j*, *f_ki_* represents the concentration matrix of element *i* in source *k*, *e_i_*_j_ represents the residual matrix, *Q* represents the objective function for solving the optimal g and *f* under non-negative restrictions (*Q* values close to the degree of freedom of the data set indicate a better fit), *U* represents the uncertainty, *MDL* represents the detection limit, *δ* represents the relative standard deviation, and *c* represents the concentration of the tested element.

### 2.5. Health Risk Assessment of HMs

#### 2.5.1. Exposure Analysis

The exposure level of HMs was assessed on the basis of the chronic daily intake (CDI, mg/(kg·d)) of dust in humans [85,86]. The US EPA health risk model was used in this research, and the corresponding parameters were localized with reference to the related research results in China in order to quantify the average daily exposure of HMs from USD to children and adults via three routes, namely ingestion, inhalation, and dermal contact. The calculation Equations are listed in Equations (6) and (9):

(6) $${\mathrm{CDI}}_{\mathrm{ingest}}=\left(C_{i}\times \mathrm{IngR}\times \mathrm{CF}\times \mathrm{EF}\times \mathrm{ED}\right)\times {\left(\mathrm{BW}\times \mathrm{AT}\right)}^{-1}$$

(7) $${\mathrm{CDI}}_{\mathrm{inhale}}=\left(C_{i}\times \mathrm{InhR}\times \mathrm{EF}\times \mathrm{ED}\right)\times {\left(\mathrm{PEF}\times \mathrm{BW}\times \mathrm{AT}\right)}^{-1}$$

(8) $${\mathrm{CDI}}_{\mathrm{dermal}}=\left(C_{i}\times \mathrm{SA}\times \mathrm{AF}\times \mathrm{ABS}\times \mathrm{EF}\times \mathrm{ED}\times \mathrm{CF}\right)\times {\left(\mathrm{BW}\times \mathrm{AT}\right)}^{-1}$$

(9) $${\mathrm{CDI}}_{\mathrm{total}}={\mathrm{CDI}}_{\mathrm{ingest}}+{\mathrm{CDI}}_{\mathrm{inhale}}+{\mathrm{CDI}}_{\mathrm{dermal}}$$

where CDI_ingest_, CDI_inhale_, and CDI_dermal_ denote the average daily exposure dose by ingestion, inhalation, and dermal contact routes, respectively. CDI_total_ denotes the total average daily exposure, *C_i_* denotes the measured concentration of HM, and the parameters and their meanings are listed in Table 1.

#### 2.5.2. Non-Carcinogenic Risk Assessment

The hazard quotient (HQ) was used to calculate the magnitude of the potential non-carcinogenic risk of individual HMs to humans. The hazard quotients of all HMs denote the non-carcinogenic hazard index (HI), which was calculated as follows:

(10) $${\mathrm{HQ}}_{ij}={\mathrm{CDI}}_{ij}/{\mathrm{RfD}}_{ij}$$

(11) $$\mathrm{HI}=\sum {\mathrm{HQ}}_{ij}={\mathrm{HQ}}_{\mathrm{ingest}}+{\mathrm{HQ}}_{\mathrm{inhale}}+{\mathrm{HQ}}_{\mathrm{dermal}}$$

where HQ*_ij_* denotes the non-carcinogenic risk quotient of element *i* under the *j*th exposure pathway. An HQ or HI < 1 indicates that the potential non-carcinogenic risk of HMs to humans is negligible, and an HQ or HI ≥ 1 indicates that HMs may pose non-carcinogenic risks to humans [92].

#### 2.5.3. Carcinogenic Risk Assessment

According to the classification list developed by the International Agency for Research on Cancer (IARC) [96], carcinogenic elements include Ni, As, and Cd. Therefore, their carcinogenic risk was quantitatively assessed using Equations (12) and (13):

(12) $$\mathrm{HI}=\sum {\mathrm{HQ}}_{ij}={\mathrm{HQ}}_{\mathrm{ingest}}+{\mathrm{HQ}}_{\mathrm{inhale}}+{\mathrm{HQ}}_{\mathrm{dermal}}$$

(13) $$\mathrm{TCR}=\sum {\mathrm{CR}}_{ij}={\mathrm{CR}}_{\mathrm{ingest}}+{\mathrm{CR}}_{\mathrm{inhale}}+{\mathrm{CR}}_{\mathrm{dermal}}$$

where CR*_ij_* denotes the carcinogenic risk quotient of element *i* under the *j*th exposure pathway, SF, CR, and TCR denote the carcinogenic slope coefficient, carcinogenic risk quotient, and the total carcinogenic risk, respectively. If TCR < 10^−6^, it means that HMs produce negligible health risks to humans. A TCR > 10^−4^ means that HMs have caused potential carcinogenic risks to humans, and if 10^−6^ ≤ TCR ≤ 10^−4^, this means that HMs have caused a risk to societal stability and human health that is acceptable or tolerated [97]. The RfD and SF were determined based on the relevant research results [93,98], as shown in Table 2.

## 3. Results and Discussions

### 3.1. Euclidean Distance Analysis of Impact Factors

**Figure 2:**
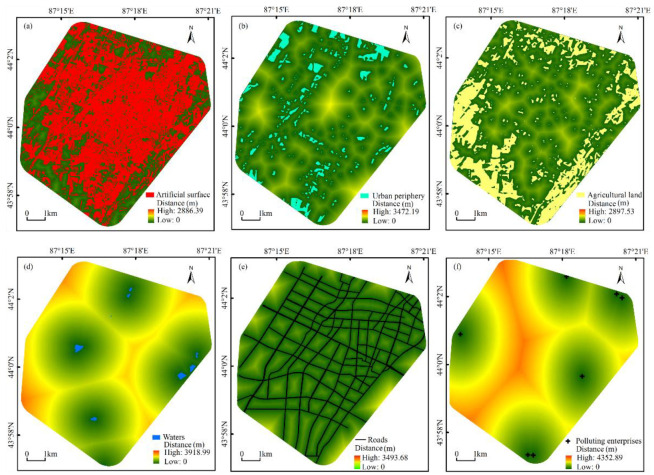
Spatial distance between sampling points and influence factors. (**a**–**f**) represent the Euclidean distances between sampling sites and artificial surfaces, urban periphery, agricultural land, waterbodies, roads, and polluting enterprises, respectively.

ArcGIS 10.2 was used to analyze the Euclidean distance of the construction land, urban green space, agricultural land, water area, roads, and polluting industries in the study area. The spatial relationship between the sampling points and these six influencing factors was drawn (Figure 2). The scale of the urban impervious surfaces is usually positively correlated with the intensity of human activities. In highly developed urban areas, impervious surfaces account for 70% of the total surface [99], and the urban river water quality is impaired when urban imperviousness reaches 12%, and more severely when imperviousness reaches 30% [25]. Therefore, the distance between the sampling point and the artificial surface can reflect the effect of human activities on USD. The urban periphery has the functions of soil fixation, dust suppression, dust absorption, and air purification. Generally, the closer the sampling point is to the urban periphery, the weaker the impact of human activities. Pollutants from fertilizers, herbicides, and pesticides used in agricultural production activities remain in the soil for a long time, which is an essential factor affecting the quality of the soil environment. The distance between the sampling point and the agricultural land can reflect the impact of pollutants released in agricultural production activities on USD. Water bodies form in relatively low-lying areas and they determine the migration direction and rate of USD in the water environment. In addition, HMs continuously accumulate in water bodies through a surface runoff, such that they eventually pollute local water bodies and pose threats to aquatic organisms [100]. Therefore, the distance between sampling sites and water bodies can reflect the impact of USD on the urban eco-environment [101]. Road density and traffic volume are essential factors influencing the concentration of HMs in USD. The distance between the sampling site and roads can reflect the influence of traffic emission activities on USD [102]. As a high-energy-consuming production unit in the city, polluting industries can adversely affect the eco-environment of the surrounding areas while maintaining production on a large scale. Polluting industries in the study area are mainly high-energy-consuming enterprises such as urban heating, the power supply, building materials production, and machinery processing and manufacturing. The distance between sampling points and polluting industries can reflect the impact of industrial activities on USD.

### 3.2. Concentration Characteristics of HMs

As shown in Table 3, the concentration ranges (average) of Pb, Ni, As, Cd, Hg, and Cu in USD were 19.0–104.0 (46.83), 20.0–66.0 (26.35), 7.82–12.70 (9.92), 0.14–0.31 (0.21), 0.023–0.162 (0.047), and 30.0–101.0 (59.33) mg/kg. The Pb, Ni, As, Cd, Hg, and Cu concentrations were 2.41, 0.99, 0.89, 1.71, 2.76, and 2.22 times higher than the corresponding soil background values in Xinjiang [103]. In addition, 26.92% of Ni and 11.54% of As exceeded their background values, indicating that the six elements were enriched to varying degrees. In addition, the skewness coefficients of the HM concentrations were all greater than 0, and the average concentrations were greater than their median values, which indicated that the distribution of HMs in the study area was positively skewed. Overall, the distribution type of the HM concentration data was long-tailed on the right, with large outliers.

According to the grading criteria of the coefficient of variation (CV) and the calculated CVs of the analyzed HMs [83], Pb and Hg were highly variable (CV > 36%), indicating that the concentration of each element varies significantly across the sampling sites, and their sources may be mainly influenced by anthropogenic activities. Meanwhile, Ni and Cu were moderately variable (16% < CV ≤ 36%), which indicates that the elements were influenced by natural and anthropogenic sources. It is noteworthy that 73.08% of the sample points for Ni were below their background values, but its skewness coefficient was as high as 4.07. This indicates the existence of some prominent point sources of pollution. The average concentration of As was lower than its background value and exhibited a low variability (CV < 16%), suggesting that As is dominated by natural sources.

### 3.3. Pollution Assessment of HMls

**Figure 3:**
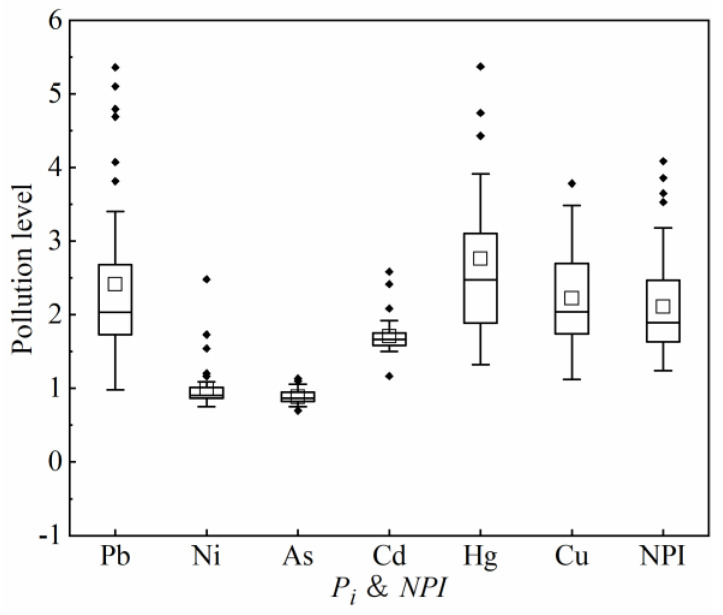
Box plots of pollution levels of heavy metals.

*P_i_* and *NPI* are commonly used to quantify the degree of HM contamination risk in USD. In Figure 3, the average values of *P_i_* followed the order of Hg > Pb > Cu > Cd > Ni > As, with higher *P_i_* values indicating greater levels of contamination. According to the grading criteria, the investigated USD was moderately polluted by Hg (*P_i_* = 2.76), Pb (*P_i_* = 2.41), and Cu (*P_i_* = 2.22), and lightly polluted by Cd (*P_i_* = 1.70). Pollution was not observed for the remaining elements, Ni (*P_i_* = 0.99) and As (*P_i_* = 0.88). The values of NPI ranged from 1.36 to 7.17, with an average value of 2.57, corresponding to the moderate pollution level.

In contrast, on the basis of the maximum *P_i_* and *NPI* values, USD was heavily polluted by Hg (*P_i_* = 9.53), Pb (*P_i_* = 5.36), and Cu (*P_i_* = 3.78), moderately polluted by Cd (*P_i_* = 1.70), and lightly polluted by As (*P_i_* = 1.13). The maximum value of *NPI* was 7.17 at the heavy pollution level. This indicated that Hg, Pb, and Cu are the primary pollutants in the USD of the study area. In addition, the value of *P_i_* had a high CV and standard deviation, indicating that the pollution levels of Hg, Pb, and Cu vary significantly across the sampling sites. Overall, the HMs in the USD of the study area were probably derived from anthropogenic sources. Therefore, monitoring and risk control of these three elements should be increased during urban development and construction in Changji city.

### 3.4. Spatial Distribution of HM Concentrations and Pollution Levels

**Figure 4:**
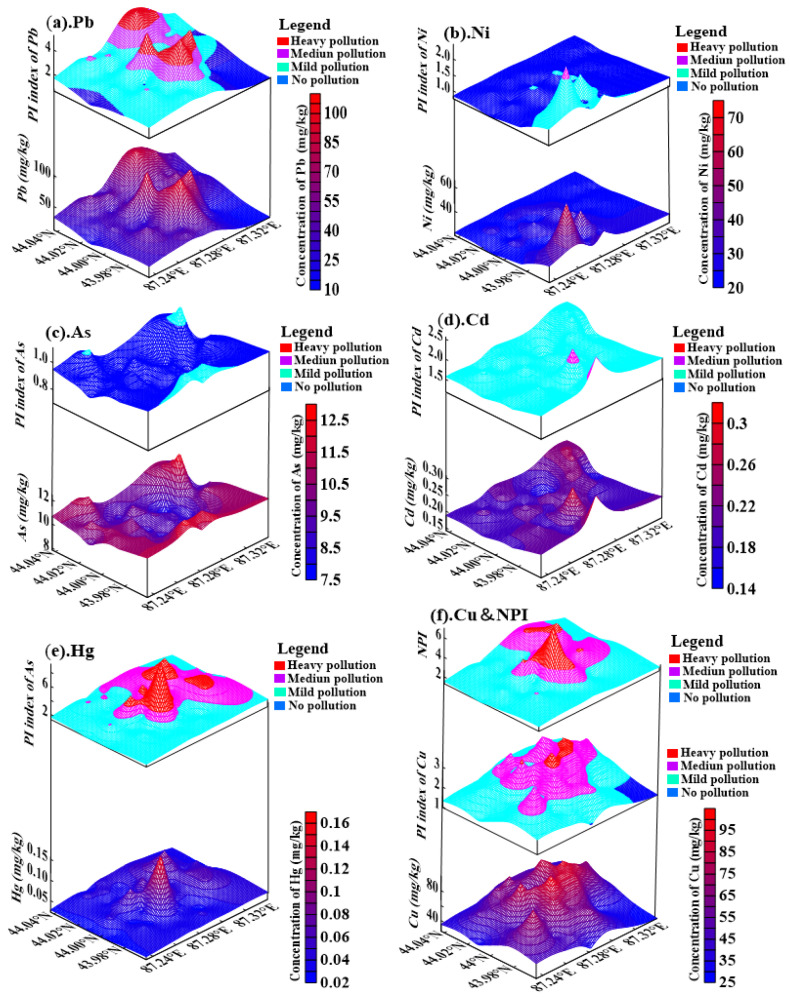
Spatial distributions of metal concentration and pollution grade.

Analyzing the spatial distribution characteristics of HMs is an effective approach to identify the sources of contaminants and high-pollution hotspots [97]. Spatial three-dimensional (3D) maps of the concentrations of Pb, Ni, As, Cd, Hg, and Cu, and their pollution levels were drawn using the Kriging interpolation method (Figure 4). Overall, the spatial distribution patterns of the HM concentrations and their pollution levels were found to be substantially heterogeneous. Hg was the largest pollutant in the study area, and in the 3D map, Hg pollution was mainly observed in the central part and the northeastern industrial area of the study area, where many activities such as the heating and power supply for the city are concentrated. Pb and Cu were the second and third largest pollutants in the study area. Pb and Cu pollution were mainly observed in the city center, which is associated with a high road density, traffic flow, and vehicle start–stop frequency [6,29]. As was the smallest pollutant in the study area. Its concentration exhibited a “basin”-shaped distribution pattern in the middle of the study area and the concentration was high in the surrounding areas. It is noteworthy that the sampling sites with As concentrations exceeding the background values of the Xinjiang soils were all near agricultural land. In addition, the spatial distribution pattern of As was similar to that of the agricultural land in the study area (Figure 2c). This suggests that agricultural activities may influence the enrichment of As elements. The areas of high values of Ni and Cd concentrations were mainly located in the electrical industrial area and the mechanical industrial area (Figure 2f), and industrial dust emitted from coal combustion, fuel oil, metal plating, and bearing production in factories contained Ni and Cd [104,105]. The spatial distribution pattern of *NPI* was similar to that of Hg, Pb, and Cu, which indicates that the spatial distribution pattern of *NPI* was mainly influenced by these elements. According to the field research and data analysis of the study area, high Pb, Cu, and Hg concentrations in USD mainly occur in the city center near the main urban roads, residential and commercial areas, and in the northeastern industrial area, which indicates that transportation, industrial emissions, and commercial activities may be the main sources of HMs in the USD of the study area [6,106,107]. In general, these elements in the USD of the study area were affected by human activities to varying degrees.

### 3.5. Non-Carcinogenic Risk of HMs

The US EPA health risk model was used to assess the potential health risk of HMs in the study area. Taking into account the physiological and behavioral differences between children and adults, the hazard quotient (HQ) and hazard index (HI) of six HMs on children and adults via ingestion, inhalation, and skin contact pathways were assessed. The HQ values of HMs for children and adults are ranked in Table 4 as HQ_As_ > HQ_Pb_ > HQ_Cu_ > HQ_Ni_ > HQ_Cd_ > HQ_Hg_. The HQ of As and Pb were higher than those of other elements, and they accounted for 65.85% and 27.44% of HI for children, respectively, compared to 65.71% and 27.66% of HI for adults, respectively. Therefore, As and Pb pose the largest major non-carcinogenic health risks in the study area. In terms of the exposure pathways, the average values of the HQ followed the order HQ_ingest_ > HQ_dermal_ > HQ_inhale_. This indicates that unconscious ingestion was the main pathway of exposure to non-carcinogenic health risks. The total HI for children (0.492) was 6.15 times higher than that for adults. This can be explained by the fact that children’s hemoglobin is more sensitive to HMs and they absorb them at a much faster rate than adults [40,108]. According to these findings, HMs in the study area pose much higher potential health risks to children than to adults. However, according to the health risk classification criteria, the values of the HQ and HI for both children and adults were lower than 1, which means that the HMs do not pose non-carcinogenic risks to human health.

### 3.6. Carcinogenic Risk of HMs

According to the classification list developed by IARC [96], Ni, As, and Cd are considered carcinogenic elements. Therefore, the carcinogenic risk (CR) and the total carcinogenic risk (TCR) of these elements for children and adults were evaluated in this research. As shown in Table 5, the CR of As was the highest in children and adults, followed by Ni, then Cd. The contribution of the CR of As to the TCR was higher than 99%. Therefore, As is the largest carcinogenic risk element in the study area. Overall, the potential carcinogenic risk from the three exposure routes followed the order: ingestion > dermal contact > inhalation. The TCR to children (1.25 × 10^−5^) was 11.79 times higher than that to adults (1.06 × 10^−6^), indicating that children are more sensitive to carcinogenic risks than adults. According to the classification standard of the TCR, the potential carcinogenic risk of HMs to children is acceptable or tolerated in the study area, but the health risk to adults can be ignored. Based on the results discussed above, As and Pb were identified as priority control elements in the study area owing to their toxicity and high potential health risk.

### 3.7. Spatial Distribution of Health Risks

**Figure 5:**
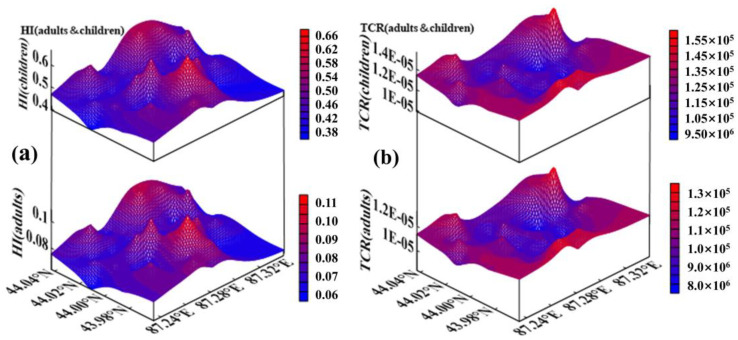
Spatial distributions of HI and TCR for children and adults. (**a**) represents the hazard index (HI) for children and adults; (**b**) represents total cancer risk (TCR) for children and adults.

The hotspots of HI for children and adults were mainly distributed in the central and northeastern regions of the study area (Figure 5a), and their spatial distribution patterns were mainly influenced by the concentration of Pb and As. The central part of the study area is the urban center with the highest traffic volume. In addition, there are several mechanical and electrical industrial parks in the northeastern part of the study area [109]. Hou et al. [110] reported on surface dust heavy metals in China for the last 10 years and found that traffic emissions were the largest source of heavy metals in 79% of the surveyed cities. This is mainly related to metallic corrosion, the abrasion of brake pads, and the leakage of a lead–acid battery [111,112,113]. Meanwhile, the spatial distribution of the TCR in children and adults showed a “basin”-shaped distribution pattern (Figure 5b). This was mainly determined by the spatial distribution characteristics of As in the study area. Soil is an important component of USD, and HMs enriched in soil enter the urban surface through resuspension. In China, approximately 10 million hectares of arable land have been polluted, and about 12 million tons of grain are contaminated each year by HMs in soil [114,115]. Studies have shown that agricultural soils in many countries have high concentrations of As [116,117]. Arsenic emissions from agricultural production activities are one of the primary sources of As in USD [118,119]. The study area is surrounded by a large area of irrigated farmland (Figure 2c), and the application of pesticides and fertilizers containing arsenic is a major contributor to the accumulation of As concentrations in farmland soils. The hazard level of HMs is mainly related to their total amount and bioavailability. Research shows that adding K_2_HPO_4_ and Na_2_SiO_3_ to sprinklers can effectively inhibit the pollution of raised dust and HMs [120]. Therefore, increasing the frequency of cleaning and sprinkling of urban roads will help protect the health of urban residents.

### 3.8. Sources of HMs

#### 3.8.1. Identification of Sources of HMs

**Figure 6:**
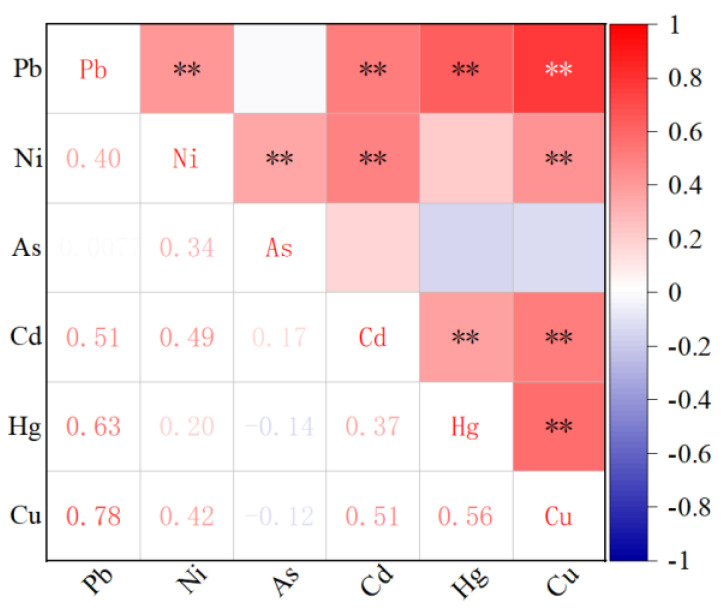
Correlation heat map of heavy metals. ** represents the significance level of *p*-value < 0.05.

HMs in USD are influenced by natural and anthropogenic sources such as soil parent material, industrial emissions, transportation, and domestic activities [19,20]. Correlation analysis and cluster analysis are often widely used to identify the pollution sources of HMs [121]. The correlation coefficients of the investigated HMs are shown in Figure 6. Pb–Cu, Ni–Cd, and Hg–Cu showed extremely significant positive correlations, indicating that these elements have a strong homogeneity. Meanwhile, Pb–Cd, Ni–Cu, and Cd–Cu showed a significant positive correlation, which means that these elements may have common or similar sources. In addition, the correlation between As and the other five elements was not statistically significant (*p* > 0.05), indicating that its primary source differed from the other elements.

**Figure 7:**
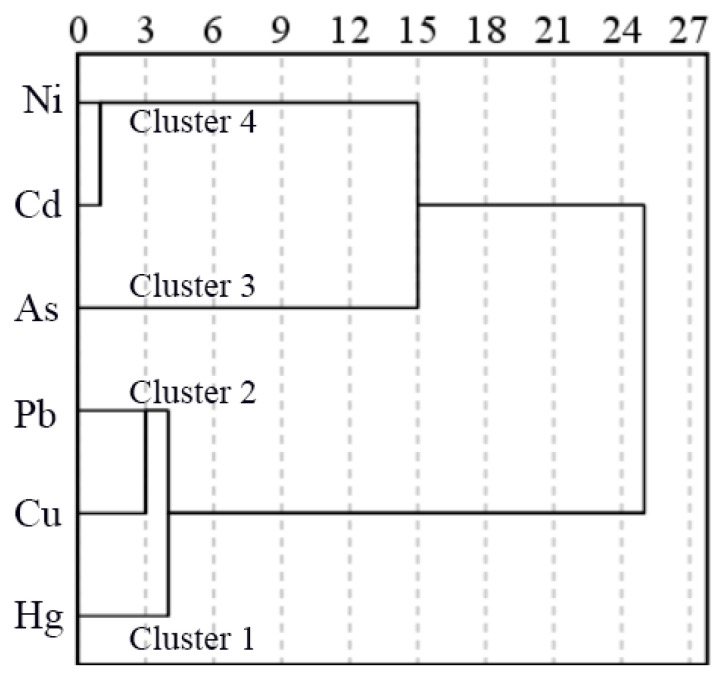
Hierarchical dendrogram of heavy metals was obtained using Ward’s clustering.

In this study, based on Ward’s method, the cluster pedigree of HMs in the USD of the study area was drawn (Figure 7). According to the cluster analysis, the six elements were grouped into four clusters (referring to three height distances): Cluster 1 with Hg, Cluster 2 with Cu and Pb, Cluster 3 with As, and Cluster 4 with Cd and Ni. In general, HMs that are divided into the same cluster indicate that they may share common sources.

The concentrations of Hg in Cluster 1 were all higher than the corresponding background values, and their hotspots were mainly located in the central and northeastern parts of the study area where traffic flow and commercial activities were most frequent. Pb and Cu in Cluster 2 showed an extremely significant positive correlation. The high value area of their concentration is mainly distributed near road intersections and main and secondary trunk roads. The concentration of As in cluster 3 was lower than the corresponding background values, and its sample concentration showed a narrow variability. In addition, the spatial distribution characteristics of As concentration were quite different from those of the other five elements. This shows that the main source of As was different from those of other elements. In cluster 4, Cd and Ni showed an extremely significant positive correlation at the 0.01 level, and areas with high Cd and Ni concentrations were mainly distributed in the southern industrial region of the study area.

#### 3.8.2. Analysis of HM Sources Based on PMF

**Figure 8:**
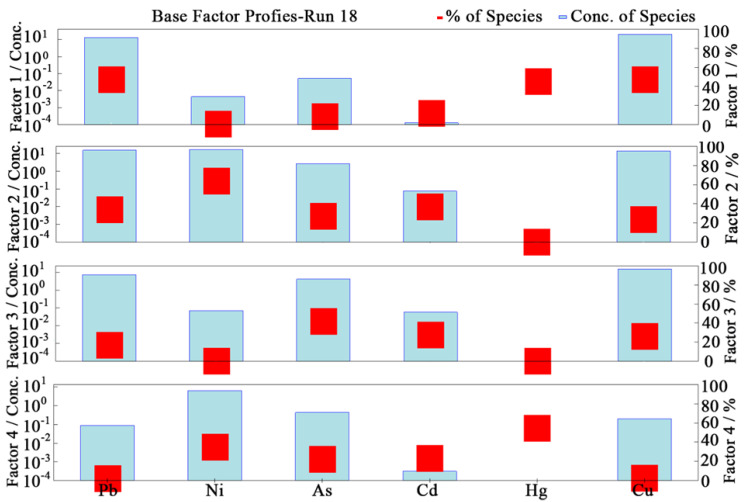
Source composition and source contribution rate of heavy metals based on positive matrix factorization.

The results of the analysis based on the PMF model are shown in Figure 8. Factor 1 was the largest contributor to Pb and Cu, indicating that Pb and Cu mainly originated from pollution source 1. The 3D hotspot maps of HM concentrations (Figure 4) showed nearly identical distributions of Pb and Cu. In addition, the correlation analysis (Figure 6) and cluster analysis (Figure 7) showed that Pb and Cu share the main pollution sources. The above analysis proves the accuracy of the analytical results of the PMF model. Similarly, the results of the PMF analysis indicated that Ni and Cd originated from pollution source 2, As from pollution source 3, and Hg from pollution source 4.

Factor 1 was the largest contributor to Pb (47.2%) and Cu (47.4%) among the four sources. The spatial distribution characteristics of Pb and Cu concentrations were nearly identical, and their hotspots were mainly distributed in the city center. From the previous discussion, it can be inferred that the central part of the study area has the largest area of urban artificial surface, and the highest road density and traffic flow. Automobiles are the main source of Pb pollution in the urban eco-environment [122]. Pb has been added to fuel as well as car components such as batteries, wheel rims, wheel balancing weights, and aluminum alloys. Pb concentrations in lead–acid batteries in private automobiles and trucks are approximately 10 kg and 12.5 kg, respectively, accounting for more than 90% of a vehicle’s total lead mass [25,123]. Nriagu [124] reported that leaded gasoline has been in use for half a century, and the total amount of lead released into the atmosphere was about 7 million tons in the United States. In 2000, the release of Cu from brake wear in Europe was approximately 2400 tons, accounting for 48% of all sources of Cu [125]. Davis et al. [126] reported that 48% of Cu in urban stormwater runoff was released from brake wear. In addition, previous studies have shown that Pb and Cu in USD mainly originate from transportation activity [127,128,129,130]. Therefore, Factor 1 denotes the source of transportation emissions.

Factor 2 was mainly defined by Ni (64.1%) and Cd (37.2%). The hotspots of Ni and Cd concentrations were mainly located near electrical and mechanical industrial areas in the southern part of the study area. The sources of Cd in USD are complex and mainly related to urban anthropogenic activities such as urban multi-source atmospheric emission, machining, and metal electroplating. In addition, Cd and Ni are reflected in the industrial dust emitted from coal-fired, oil-fired, and bearing production in factories [104,105]. Hence, factor 2 denotes the industrial sources.

The element with the highest loading in Factor 3 was As (41.8%), and its average concentration was lower than the corresponding background value. As showed a low variability among the sampling sites. In addition, As showed a relatively uniform distribution in the study area. Moreover, As was found to be primarily impacted by a combination of the geochemical composition of the parent material of the soil and geogenic processes [20]. Therefore, factor 3 denotes the natural sources.

Factor 4 was related to a strong loading of Hg (54.8%) that might have originated from a single pollution source. This proportion was significantly higher than that of the other elements. Therefore, Hg can be considered as the signature element of factor 4. The results of the pollution evaluation show that Hg was the largest pollutant in the study area (Figure 3). The hotspots of Hg concentration are mainly distributed in the coal-fired northeast industrial area for urban heating and power supply and the city center with the most frequent commercial and domestic activities. In 2019, there were 46 large-scale industrial enterprises in the study area whose energy structure was dominated by coal combustion. In addition, Hg is a volatile component of coal, and nearly 100% of Hg enters the flue gas during the combustion process, and dust collectors have a high dust removal efficiency for oxidized mercury (Hg^2+^) and particulate Hg [131,132]. However, elemental mercury (Hg^0^) is extremely insoluble in water and difficult to absorb by dust collectors, thus leading to its escape into the atmosphere [133]. Abliz et al. [134] reported that the main sources of Hg pollution are coal combustion industries and coal gangue dumps. The level of Hg in the environment has increased primarily as a result of industrialization and its byproducts [67,135]. With the second-largest gas reserves in the world, Asaluyeh has the largest oil and gas reserves in Iran. Its petrochemicals contribute significantly to high Hg levels in USD [136,137]. In the study area, heating is facilitated by coal combustion for up to 6 months per year, which is an important reason for the gradual accumulation of Hg in the urban environment. Thus, factor 4 reflects anthropogenic sources, which is primarily coal consumption.

According to the fingerprints of HM sources, the total contribution of the four sources was found to follow the order: industrial emissions (31.08%) > traffic emissions (26.80%) > coal combustion (23.31%) > natural sources (18.81%). Overall, anthropogenic activities were the dominant factor leading to the accumulation of HMs in the USD of in the study area.

## 4. Conclusions

In this research, 52 USD samples were collected from Changji city, NW China, and the concentrations of Pb, Ni, As, Cd, Hg, and Cu were determined. In brief, the concentrations, pollution levels, spatial distribution, primary sources, and health hazards of these heavy metals were investigated. The average concentrations of Pb, Ni, As, Cd, Hg, and Cu were 2.41, 0.99, 0.89, 1.71, 2.76, and 2.22 times higher than the corresponding background values. According to the results of the pollution assessment, USD in the study area was moderately polluted by Hg, Pb, and Cu, mildly polluted by Cd, and not polluted by Ni and As. The spatial distribution analysis revealed that the hotspots of Pb, Hg, and Cu were mainly located in the city center and northeastern industrial areas. Hotspots of Ni and Cd were mainly located in the electrical and mechanical industrial areas. The route with the highest potential for human exposure to HMs was ingestion, and As and Pb were the main non-carcinogenic elements. Furthermore, As was the largest contributor to carcinogenic risks, although its level of potential health risks is tolerable. The correlation analysis, cluster analysis, and PMF model analysis indicated that the HMs in the USD of the study area mainly originated from four pollution sources. The analytical results based on the PMF model showed that the total contribution rates of industrial emissions, traffic emissions, coal combustion, and natural sources of HMs were 31.08%, 26.80%, 23.31%, and 18.81%, respectively. The results also showed that anthropogenic sources are the primary source of HMs, and thus special attention should be paid to reducing pollution sources and controlling the risk to the environment and human health.

## Figures and Tables

**Table 1:** The exposure parameters are used to estimate chronic daily intake.

Indicators	Parameters	Meaning (Units)	Children	Adult	Reference
Exposure factor	EF	Exposure frequency (d/a)	350	350	[87]
	ED	Exposure duration (year)	6	30	[88]
	AT_nc_	Average exposure time for non-cancer (d)	365 × ED	365 × ED	[89]
	AT_ca_	Average exposure time for cancer (d)	365 × 70	365 × 70	[89]
	BW	Average body weight (kg)	21.2	62.4	[90,91]
	CF	Unit conversion factor (kg/mg)	1 × 10^−6^	1 × 10^−6^	[92]
Ingestion	IngR	Consumption rate of dusts (mg/d)	200	100	[92]
Inhalation	InhR	Dust inhalation rate (m^3^/d)	7.5	16.2	[93]
	PEF	Particulate emission factor (m^3^/kg)	1.36 × 10^9^	1.36 × 10^9^	[94]
Skin contact	AF	Skin adherence factor (mg/(cm^2^/d))	0.20	0.07	[92]
	SA	Exposed skin area (cm^2^)	899	1600	[95]
	ABS	Dermal absorption factor (unitless)	Pb = Ni = Hg = Cu = 0.01; As = 0.03; Cd = 0.005		[38]

**Table 2:** Reference dose and slope factor for three exposure pathways of heavy metals.

Elements	RfD (mg/(kg·d)			SF/(mg/kg·d)^−1^		
	Ingestion	Inhalation	Dermal Contact	Ingestion	Inhalation	Dermal Contact
Pb	0.0035	0.00352	0.000525	-	-	-
Ni	0.020	0.0206	0.0054	-	0.84	-
As	0.0003	0.000123	0.0003	1.50	0.0043	1.50
Cd	0.001	0.001	1.0 × 10^−5^	-	6.30	-
Hg	0.0003	0.0003	2.4 × 10^−5^	-	-	-
Cu	0.04	0.0402	0.012	-	-	-

**Table 3:** Descriptive statistics of heavy metals concentrations in urban surface dusts (*n* = 52).

Items	Pb	Ni	As	Cd	Hg	Cu
Minimum/(mg/kg)	19.0	20.0	7.82	0.14	0.023	30.0
Maximum/(mg/kg)	104.0	66.0	12.70	0.31	0.162	101.0
Average/(mg/kg)	46.83	26.35	9.92	0.21	0.047	59.33
Median/(mg/kg)	39.50	24.00	9.72	0.20	0.0421	54.50
St. D/(mg/kg)	19.71	7.13	1.01	0.03	0.024	17.62
CV/%	42.09	27.06	10.18	14.29	51.06	29.69
Skewness	1.36	4.07	0.64	1.74	2.72	0.47
Kurtosis	1.42	19.70	0.34	5.84	10.00	−0.63
Background value/(mg/kg)	19.40	26.60	11.20	0.12	0.017	26.70

**Table 4:** The non-carcinogenic risk indexes of heavy metals in urban surface dust.

	HQ_ingest_		HQ_inhale_		HQ_dermal_		HQ		HI	
	Children	Adults	Children	Adults	Children	Adults	Children	Adults	Children	Adults
Pb	1.28 × 10^−1^	2.06 × 10^−2^	3.50 × 10^−6^	2.44 × 10^−6^	7.67 × 10^−3^	1.54 × 10^−3^	1.35 × 10^−1^	2.21 × 10^−2^	4.92 × 10^−1^	7.99 × 10^−2^
Ni	1.26 × 10^−2^	2.02 × 10^−3^	3.37 × 10^−7^	2.34 × 10^−7^	4.19 × 10^−4^	8.40 × 10^−5^	1.30 × 10^−2^	2.11 × 10^−3^		
As	3.16 × 10^−1^	5.08 × 10^−2^	2.12 × 10^−5^	1.48 × 10^−5^	8.52 × 10^−3^	1.71 × 10^−3^	3.24 × 10^−1^	5.25 × 10^−2^		
Cd	1.96 × 10^−3^	3.15 × 10^−4^	5.40 × 10^−8^	3.76 × 10^−8^	8.81 × 10^−4^	1.77 × 10^−4^	2.84 × 10^−3^	4.92 × 10^−4^		
Hg	1.50 × 10^−3^	2.41 × 10^−4^	4.12 × 10^−8^	2.87 × 10^−8^	1.68 × 10^−4^	3.37 × 10^−5^	1.66 × 10^−3^	2.74 × 10^−4^		
Cu	1.42 × 10^−2^	2.28 × 10^−3^	3.89 × 10^−7^	2.70 × 10^−7^	4.24 × 10^−4^	8.51 × 10^−5^	1.46 × 10^−2^	2.36 × 10^−3^		

**Table 5:** The carcinogenic risk indexes of heavy metals in urban surface dust.

	CR_ingest_		CR_inhale_		CR_dermal_		CR		TCR	
	Children	Adults	Children	Adults	Children	Adults	Children	Adults	Children	Adults
Ni	-	-	5.00 × 10^−10^	1.74 × 10^−9^	-	-	5.00 × 10^−10^	1.74 × 10^−9^	1.25 × 10^−5^	1.06 × 10^−5^
As	1.22 × 10^−5^	9.80 × 10^−6^	9.63 × 10^−13^	3.35 × 10^−12^	3.29 × 10^−7^	7.68 × 10^−7^	1.25 × 10^−5^	1.06 × 10^−5^		
Cd	-	-	2.92 × 10^−11^	1.01 × 10^−10^	-	-	2.92 × 10^−11^	2.92 × 10^−11^		

## Data Availability

Not applicable.
